# Extension of Methane Emission Rate Distribution for Permian Basin Oil and Gas Production Infrastructure by Aerial LiDAR

**DOI:** 10.1021/acs.est.3c00229

**Published:** 2023-08-10

**Authors:** William M. Kunkel, Asa E. Carre-Burritt, Grant S. Aivazian, Nicholas C. Snow, Jacob T. Harris, Tagert S. Mueller, Peter A. Roos, Michael J. Thorpe

**Affiliations:** Bridger Photonics Incorporated, 2310 University Way Bldg 4-4, Bozeman, Montana 59715, United States

**Keywords:** CH_4_, remote sensing, detection probability, sensitivity, aggregation, point source

## Abstract

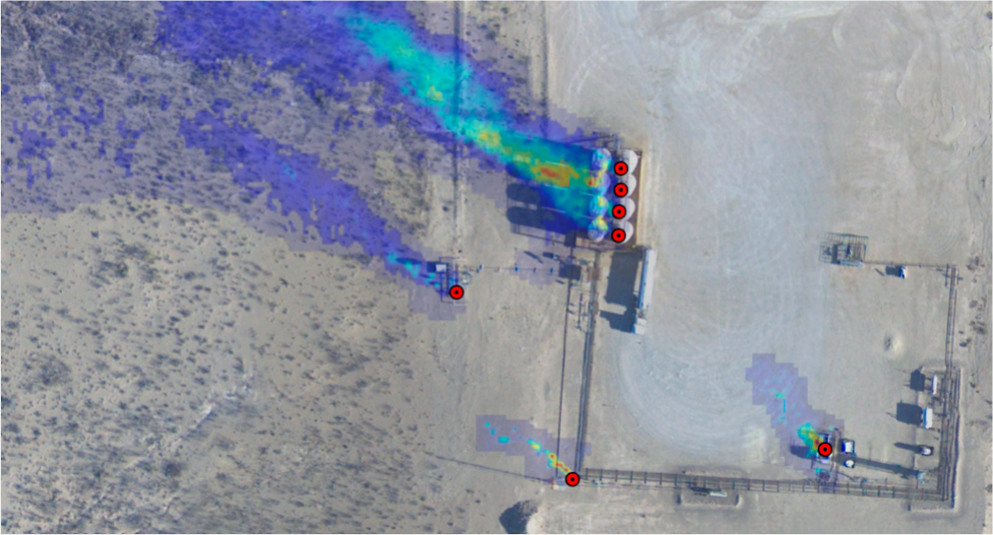

Aerial LiDAR measurements at 7474 oil and gas production facilities in the Permian Basin yield a measured methane emission rate distribution extending to the detection sensitivity of the method, 2 kg/h at 90% probability of detection (POD). Emissions are found at 38.3% of facilities scanned, a significantly higher proportion than reported in lower-sensitivity campaigns. LiDAR measurements are analyzed in combination with measurements of the heavy tail portion of the distribution (>600 kg/h) obtained from an airborne solar infrared imaging spectrometry campaign by Carbon Mapper (CM). A joint distribution is found by fitting the aligned LiDAR and CM data. By comparing the aerial samples to the joint distribution, the practical detection sensitivity of the CM 2019 campaign is found to be 280 kg/h [256, 309] (95% confidence) at 50% POD for facility-sized emission sources. With respect to the joint model distribution and its confidence interval, the LiDAR campaign is found to have measured 103.6% [93.5, 114.2%] of the total emission rate predicted by the model for equipment-sized emission sources (∼2 m diameter) with emission rates above 3 kg/h, whereas the CM 2019 campaign is found to have measured 39.7% [34.6, 45.1%] of the same quantity for facility-sized sources (150 m diameter) above 10 kg/h. The analysis is repeated with data from CM 2020–21 campaigns with similar results. The combined distributions represent a more comprehensive view of the emission rate distribution in the survey area, revealing the significance of previously underreported emission sources at rates below the detection sensitivity of some emissions monitoring campaigns.

## Abstract 2

New methane emission measurements in the Permian Basin extend the distribution of source emission rates beyond the sensitivity limits of previous studies by 2 orders of magnitude.

## Introduction

Methane is a potent greenhouse gas with a warming potential 80 times greater than that of CO_2_ in a 20 year time frame.^1^ Its current global emission rate is great enough to impact the climate significantly, with a greater contribution to global temperature rise in the first ten years after emission than CO_2_ at current emission rates of each gas.^2^ Consequently, mitigation of methane emissions is viewed as particularly important for meeting climate goals within the next decade. Economic sectors including agriculture, waste disposal, and energy are recognized as leading contributors to anthropogenic methane emissions, representing domains where emissions can be most meaningfully mitigated. In the oil and natural gas (O&G) industry, emissions arise from discrete infrastructure elements and associated processes that can often be addressed with targeted intervention. Mitigation involves both the detection of emission sources and follow-up with repair and/or upgrade of emitting equipment. Identifying the most important emissions drivers and tracking the efficacy of mitigation efforts is key to making emissions reductions effective and efficient.^3,4^

Broadening the view of emissions from individual sources to a distribution of sources provides a large-scale context to set meaningful mitigation goals. Past characterization of methane emission distributions has often relied on bottom-up estimates based on emission factors, such as those used for the U.S. Environmental Protection Agency’s Greenhouse Gas Reporting Program and Greenhouse Gas Inventory. These estimates aim to identify dominant emission sources at the component or equipment level but have been shown to misrepresent large-scale methane emissions distributions and the relative contribution of different elements,^3,5−8^ with the greatest discrepancies existing in the production sector.^9^ In addition, emission factors are meant to apply nationally, whereas emission intensities in fact vary regionally and mitigation is performed locally.^7,8^ To more precisely account for emissions and inform mitigation efforts, measurement campaigns have been conducted to obtain locally relevant empirical data within individual production basins throughout the United States and Canada.^10−15^

Many recent research efforts have focused on the Permian Basin because of its sizable share of U.S. O&G production, comprising 43% of domestic oil and 22% of natural gas produced annually.^16^ Two studies on 2018/2019 Permian methane emissions both estimated region-wide O&G methane intensity to be 3.7% of production,^17,18^ exceeding an estimated national average of 2.3% for the full supply chain.^7^ More recent work has provided methane intensity estimates in the range of 5–6% in 2018 and 3–4% in 2020.^19^ Aerial measurements conducted in 2019–21, coupled with simulated emission sources representing the unmeasured part of the distribution, provided a Permian Basin methane intensity estimate of 5.29%.^20^ With the exception of ref (20), these studies leveraged satellite observations for inversion modeling and mass balance calculations, which are useful in benchmarking overall emissions but lack the detection sensitivity or spatial resolution needed to identify individual methane sources and understand their relation to infrastructure elements.

To provide a more specific account of emission sources, aerial campaigns conducted by Carbon Mapper (CM)^21,22^ and a separate one reported by Chen et al.^23^ performed emissions measurements in the Permian Basin using solar infrared imaging spectrometers. The first CM campaign^21^ took place in 2019 and covered 55 000 km^2^ in the Midland and Delaware sub-basins located in Texas and New Mexico. Emission sources were localized and attributed to individual facilities. Repeated sampling of the same sources was used to evaluate emission intermittency. Highly intermittent sources (0–25% observed persistence of facility-sized sources) were responsible for 48% of all point source emissions in the sample. Further campaigns were run in the Permian Basin in 2020–21^22^ in a spatial domain partially overlapping with the 2019 campaign, with otherwise similar collection parameters.

The study by Chen et al.^23^ was focused on the New Mexico Permian and encompassed over 90% of wellheads in that region. Chen et al. compared the measured emission rate distribution from their study to CM 2019 in an overlapping spatial region and found that the CM 2019 campaign detected progressively fewer emission sources at rates below roughly 300 kg/h, while their own study observed similarly reduced detections below 100–150 kg/h. Though the decline in detected emission sources suggests that the CM 2019 data underrepresent the actual emission sources present below the detection sensitivity, the heavy tail portion of the dataset can still valuably inform models of the emission rate distribution.

For the present work, CM data are combined and compared to compiled survey data from Bridger Photonics’ first-generation Gas Mapping LiDAR (GML) sensor. Emission rates in the range of 3–300 kg/h, which are underrepresented in the CM campaigns, are detected by GML at their true frequency. In a complementary manner, the CM campaign datasets offer extensive sampling of large-rate but infrequently emitting sources. Detection data from CM and GML campaigns are joined to obtain a comprehensive view of the emission rate distribution in the survey region. Emission sources at rates observable by GML but not by CM are seen to contribute most of the total rate for the whole distribution.

## Methods

The Bridger Photonics GML instrument is an aircraft-mounted remote sensing device that maps methane concentration with coaligned dual LiDAR measurements, geospatial data through a global navigation satellite system, and aerial photography to show plume shape, identify the source of the emission, and quantify the emission rate. Coaligned range-finding and gas absorption lasers are spatially scanned in a conical pattern below the aircraft. A return signal originating from ground-based backscatter is detected at the sensor. Path-integrated gas concentration is measured using wavelength modulation spectroscopy on the 1651 nm absorption line of methane. Flux rates are found from total methane concentration integrated along the direction perpendicular to the gas flow direction, multiplied by wind speed at the measured plume height. Details of the collection platform have been described previously.^24^

For the surveys used in this paper, the GML instrument was flown at an altitude of 206 m above ground level (AGL), with a measured detection sensitivity of 0.41 kg/h per m/s wind speed at 90% probability of detection (POD)^25^ or nominally 2 kg/h at the average wind speed of 4.9 m/s in Midland, TX.^26^ Scan parameters are chosen so that the distance between LiDAR measurement points on the ground is 1 m at maximum.

The CM campaigns in this paper utilize two similar instruments called Global Airborne Observatory (GAO) and Airborne Visible/InfraRed Imaging Spectrometer-Next Generation (AVIRIS-NG) based on solar infrared spectroscopic imaging. The CM data offer extensive sampling of the heavy tail of the distribution but lower detection sensitivity and reduced spatial source resolution compared to GML. The instruments were flown at altitudes of 4.5 and 8 km^21^ AGL. High flight altitudes like these offer greater coverage rates (land area per time) but lower detection sensitivity. The performance of the CM instrument as a function of altitude has been characterized in controlled releases^27^ and modeled with a robust Bayesian approach.^28^ In this paper, campaign flights are grouped together regardless of altitude to assess campaign-specific performance rather than instrument performance in general. The campaign sensitivity is thus an average of the measurement sensitivity at the various conditions observed in the campaign, including flight altitude.

Whereas the spatial pixel size increases with altitude (CM: 3–8 m for 3–8 km flight altitude^29,30^), it is important to distinguish between pixel size and source resolution or the spatial area over which detected emissions are considered to come from the same source. An emission “source” in this paper means a set of synchronously or asynchronously detected plumes falling into a defined aggregation area, whereas “emitter” means a source smaller than the source resolution of the measurement system, inclusive of processing. In addition to limits imposed by image resolution, CM employs a 150 m diameter aggregation area to define its sources at roughly the size of a typical well pad.

**Figure 1:**
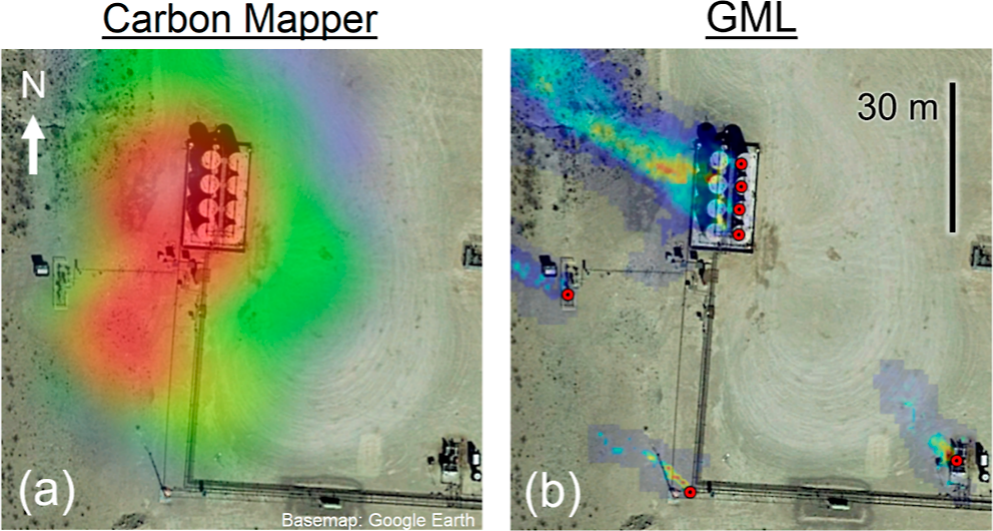
Example facility overlay with methane plume images from (a) CM 2019^31^ and (b) GML scans superimposed on satellite imagery. GML emission locations are marked by red dots. CM 2019 and GML plumes were observed on different dates; the GML image comprises plumes observed on multiple dates. GML identifies unique emission sources at an interval of 4–5 m on a tank battery (upper center; observed simultaneously).

To compare the spatial characteristics of GML and CM emission sources, consider the example plume imagery overlaid on satellite visible imagery shown in Figure 1. The same facility was observed by both AVIRIS-NG and GML on different dates. In (a), two possible gas concentration peaks are not quite distinguishable, whereas in (b), multiple GML plumes are visible. GML pins mark the localization of point sources with a precision of 2 m, which roughly corresponds to the size of typical production equipment. Asynchronous detections at these locations must be localized to within 2 m to count toward the same emission source. For CM, by contrast, all emitters within 150 m are aggregated to the same source. This tends to increase source emission rates because multiple emitters are summed to obtain the reported source rate. For comparison on an equal basis with CM, GML detections can also be aggregated to 150 m diameter groups. Cases where spatial aggregation is performed are labeled in the analysis. Further details of GML spatial aggregation are given in Section S1.

Further parameters, including data selection, measurement time frame, survey area, and scan repetitions, were considered in compiling the datasets. Details of the data compilation are given in Section S2. Statistical tests on the heavy tail part of the distribution are run to check the assumption that the GML and CM datasets sample the same distribution (Section S3).

## Results

After alignment, we combine the CM and GML datasets to obtain a joint model of the emission rate distribution. We describe results in terms of the detection density and cumulative emission rate distribution. The detection sensitivity of the CM campaign is quantified by comparing the CM detection density to the joint model function, and the share of the cumulative emission rate measured by each campaign (scaled by sample size) is also inferred by comparing it to the model. We first run the analysis on facility-sized sources (150 m diameter) and then repeat the process on single-emitter sources. This highlights differences between the distributions due to spatial aggregation to facility-sized sources. Results from the CM 2020–21 campaigns are also shown.

### Facility-Sized Aggregated (150 m) Emission Sources

As a first step to joint analysis, we establish a comparison domain supported by both the GML and CM samples. Sensitivity limits associated with each sample determine that emission sources below the full detection limit (FDL) will be detected with diminishing probability as the emission rate decreases. The POD for a given source can be rigorously characterized as a function of emission rate, wind speed, and flight altitude using controlled release data in a robust Bayesian formalism.^28^ In this work, we take a simpler approach to the analysis by restricting the emission rate domain to rates above the greater FDL of both samples. For the GML and CM samples, the limiting FDL is set by CM. We choose *x*_L_ = 600 kg/h as the effective FDL of the CM measurements. The accuracy of the declared FDL is not critical as long as it is large enough to avoid introducing observations at a significantly reduced POD. All distributions are presented in the single-scan equivalent form described in Section S2.4.1, which can be understood as a distribution on a characteristic emission rate from a single observation of a given source, subject to the detection sensitivity limits of the measurement campaign. The characteristic emission rate approximates an instantaneous source emission rate that would be observed in a single overflight; spatial aggregation and multiple overflights effectively sum and average the rate across observations of the source.

Before fitting the data to obtain a joint distribution, we run a preliminary check on the sample distributions using a hypothesis test based on the Kolmogorov–Smirnov (K–S) statistic. The test is meant to show whether the GML and CM samples differ significantly above the CM FDL, that is, in the heavy tail portion of the distribution. The outcome of the test does not contradict the assumption that GML and CM samples follow the same distribution (Section S3).

We next create a model of the distribution that represents both samples. The model density function is taken to follow a generalized lognormal distribution
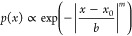
1where *x* is the base-10 logarithm of the emission rate and *x*_0_, *b*, and *m* are fit parameters, where *b* > 0 and *m* > 0. Integration over the range *x*_L_ ≤ *x* < *∞* yields the survival function
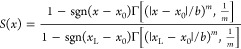
2which has been adapted to the integration range and direction so that lim_*x*→*∞*_*S*(*x*) = 0 and *S*(*x*_L_) = 1. In the special case where *m* = 2, the generalized lognormal distribution is simply lognormal. In either case, the fit parameters are jointly optimized using maximum likelihood estimation (MLE). With the joint likelihood function given in Section S4, the samples can be fit jointly below their respective FDLs. A nominal value of *x*_L_ = 3 kg/h is chosen for the GML FDL for equipment-sized sources, consistent with the sensitivity of 2 kg/h (90% POD) mentioned in the Methods section. For facility-sized sources, this is increased to *x*_L_ = 10 kg/h to avoid underestimating the FDL since spatial aggregation increases source emission rates. The MLE fitting process accounts for differences in sample size so the source densities are compared without requiring normalization based on survey size or number of overflights.

Several candidate fits are considered. The joint fit is compared to single-sample fits using lognormal and generalized lognormal forms for the density function. The purpose is to confirm that the joint fit better represents the two samples and to choose a model function that more accurately represents the two samples, particularly with respect to the “tailedness” of the distribution determined by *m* in eq 1. After obtaining fit parameters, the candidate models are assessed for the relative likelihood of information loss using the Akaike information criterion (AIC).^32^ The AIC comparison shows that the joint lognormal fit is optimal for 150 m emission sources (GML with CM 2019 or CM 2020–21), whereas a generalized lognormal model is preferred for equipment-sized emitters (GML with CM 2019; *m* = 1.619). Details of the AIC analysis are given in Section S5. The best fit parameters for all candidate models are shown in Table S1. Those from the optimal candidate provide the current best-known representation of the distribution based on the GML and CM data.

**Figure 2:**
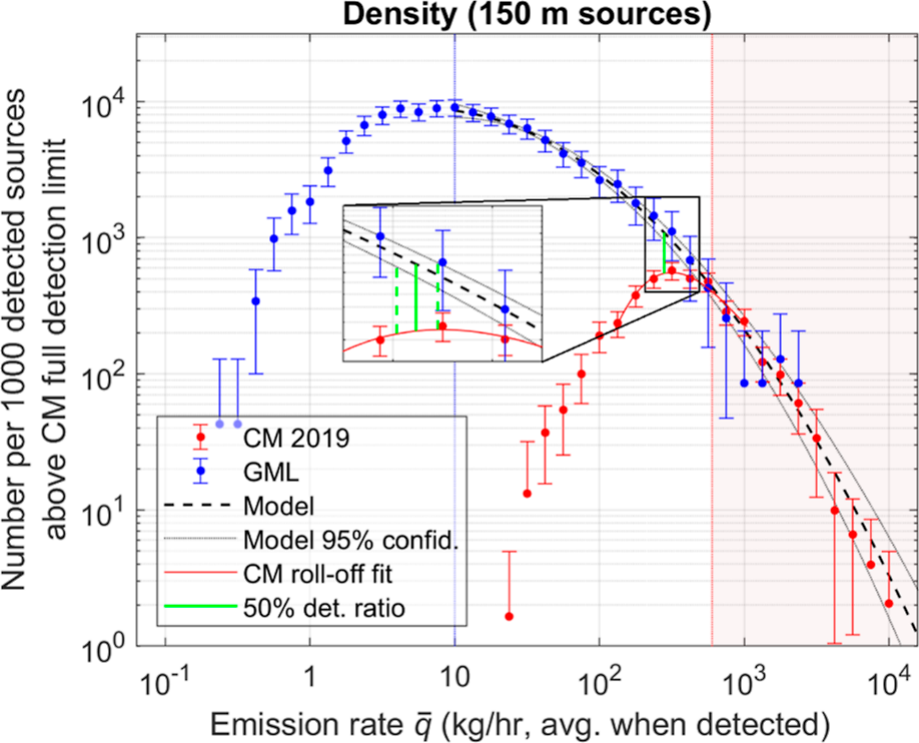
CM 2019 and GML emission source density as a function of emission rate, where sources have a 150 m diameter aggregation area. Zoomed-in view near the sensitivity limit (inset) shows the 50% detection ratio with respect to the model function and its confidence bounds. Model function follows eq 1 with *m* = 2, *x*_0_ = 0.797, and *b* = 1.140.

With fit parameters obtained from the joint likelihood analysis, the resulting density function is shown in Figure 2. Survey detections are binned by the emission rate, and the entire sample is scaled to a reference total of 1000 detected sources above the CM FDL. Error bars are placed at , where *p*_bin_ is the density value of the bin and *n*_bin_ is the count of emission sources in the bin. Confidence bounds for the model fit are calculated using the likelihood ratio (LR) method at 5% rejection. The bounds consist of the most extreme value of the distribution function at every emission rate among the locus of solutions on the rejection contour. Fit agreement and scale factors are described in Section S6.

Though the three traces in Figure 2 (CM 2019, GML, and model) agree above the CM FDL, CM detection density diminishes rapidly at emission rates below the FDL. By comparing the model to the CM detection distribution around the roll-off region using an error-weighted cubic polynomial fit of the binned data, the 50% detection ratio is placed at 280 [256, 309] kg/h, where the confidence interval (CI) is found by comparing to the 95% CI of the model fit, neglecting error in the cubic polynomial estimating the roll-off, as shown in the inset. This resulting sensitivity is considerably higher than the detection limit quoted by Cusworth et al.^21^ at 10–20 kg/h but is consistent with a previous estimate of the sensitivity in the range of 100–300 kg/h.^23^ Without compensation, reduced POD leads to a significant underrepresentation of emission sources below the sensitivity. For example, comparing the detection density of CM to GML binned data at 100 kg/h shows that emission sources at this rate are in fact 14 times more common than the CM data would suggest. The CM campaign can be expected to underestimate both the fraction of facilities with emissions and the total emission rate for the facilities surveyed because of its sensitivity limit. In fact, the CM 2019 campaign detected emissions at 1.48% of well sites, whereas the GML detected emissions at 38.3% of facilities (see Section S1).

Controlled release measurements could confirm the sensitivity findings reported in this work. Alignment of flight altitude and on-the-ground properties of the sources observed in the campaign, such as ground cover, would need to be considered. Moreover, the effects of spatial aggregation would need to be accounted for to achieve the same measure of “realized” detection sensitivity for the source definition used in the campaign.

### Equipment-Sized Emission Sources

Although the above results for 150 m sources show that emission rates less than ∼300 kg/h are underrepresented in the CM detection density, a further increase in density of lower emission rates occurs when sources are resolved to equipment size scale (∼2 m). Facility-aggregated emission rates tend to be higher than equipment rates because co-located emitters on a site count toward the same emission source. Equipment-sized source resolution tends to be more practical for both bottom-up emissions modeling and identification for leak detection and repair.

To obtain the equipment-scale emission distribution, GML detections are considered in their native resolution (∼2 m) and not aggregated to 150 m. Since CM sources are not reported at finer resolution, we instead manually filter them based on associated plume imagery^31^ to include only sources with a single point emission (see details in Section S7).

**Figure 3:**
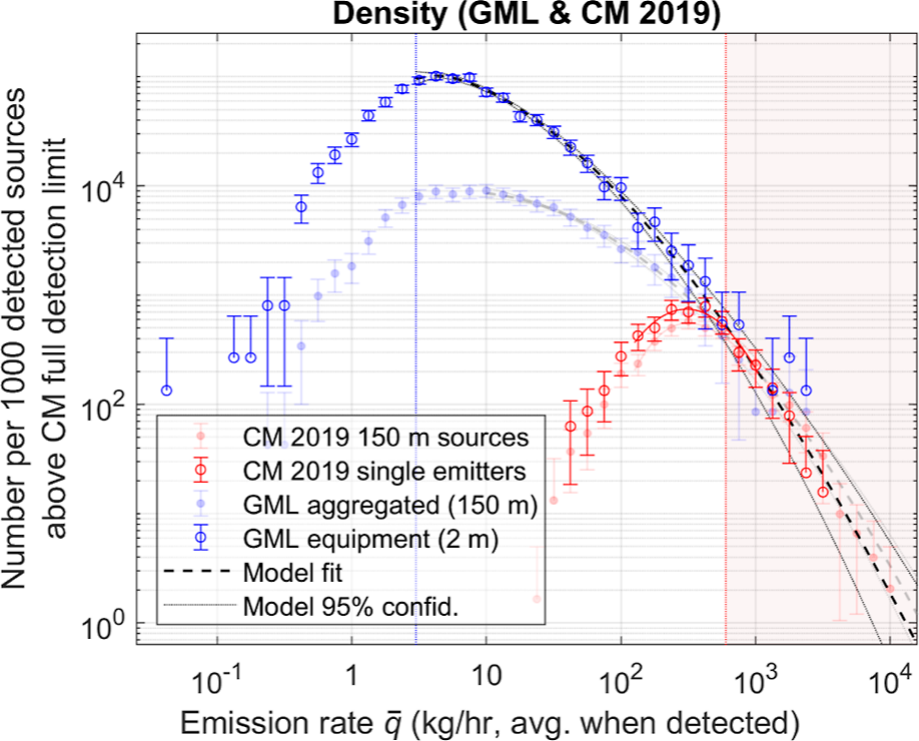
Density of detected emission sources with both 150 m aggregation diameter and no aggregation (single emitters), plotted together on the same axes. Model function for single emitter distribution follows eq 1 with *m* = 1.619, *x*_0_ = 0.629, and *b* = 0.770.

The detection density for equipment-sized sources is shown in Figure 3, with facility (150 m) detection density traces from Figure 2 reproduced for comparison. At mid-range emission rates (∼3–300 kg/h), density is significantly higher for equipment-sized sources than for facility-sized sources. For example, comparing the two GML traces at 10 kg/h shows that equipment sources at this emission rate are observed 8 times more frequently than 150 m ones. Single-emitter filters applied to the CM dataset do not appear to distort the distribution appreciably above the CM FDL. For other CM distributions (CM 2019 single emitters, CM 2020–21 150 m sources), CM detection sensitivity is assessed in a similar manner (Section S8).

### Cumulative Emission Rate Distribution

**Figure 4:**
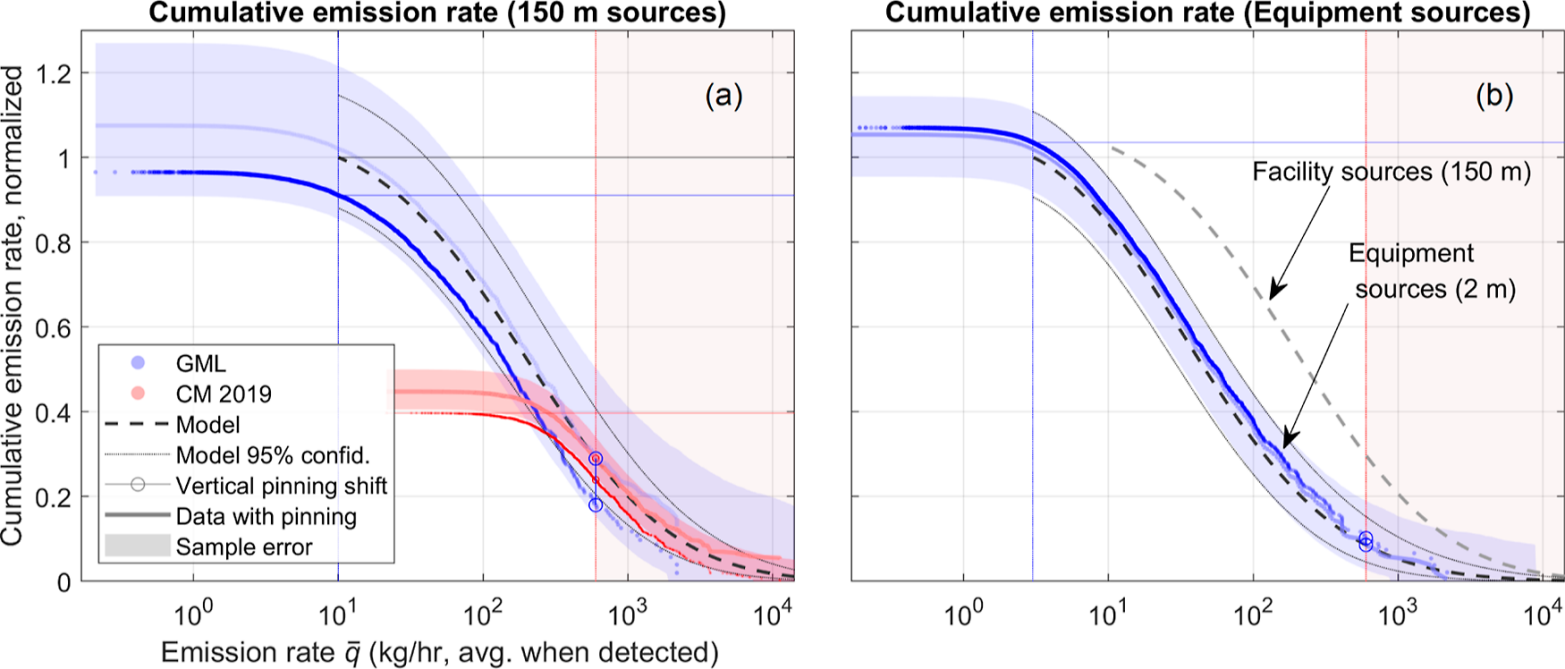
Cumulative emission rate distribution of (a) 150 m aggregated emission sources (GML and CM 2019) and (b) equipment sources (GML only). Error bounds (shaded regions) describe the predicted sample variation. Model distribution from (a) is reproduced in (b) for comparison. All traces are normalized to an equivalent campaign scale (spatial area, number of overflights). In (b), the “facility sources” model function is multiplied by the ratio of the quantity *c*(10 kg/h)/*c*(0) for each sample (see eq S2, single-scan equivalent) so that cumulative rates are comparable between traces. Vertically shifted copies of survey data pinned to the value of the model distribution at the CM FDL guide the eye to suggest the shape of the measured distribution supposing sample error above the CM FDL were suppressed.

The density function weighted by the emission rate can be integrated to yield the cumulative emission rate distributions shown in Figure 4. For measured samples, the cumulative sum is given by eq S2 (single-scan equivalent). Results for CM 2019 (150 m sources and single emitter sources) are shown in this section; cumulative emission rates for CM 2020–21 are shown in Section S9.

The expected error due to sample variation is shown in the plot. Error bounds show the 2.5 and 97.5 percentiles of the sample variation for an equivalently sized dataset with the same number of detections above the corresponding FDL, assuming that the best-fit model represents the “true” distribution. They are found by running a Monte Carlo simulation of random sets of detections drawn from the model density function (see Section S10). Sample error from sources with emission rates below each FDL is neglected, as is instrument quantification error. Sample variation in the heavy tail is responsible for much of the sample error along the entire trace. The relatively infrequently found emitters in this emission rate range have a disproportionately large impact on cumulative emission rate.

The fractional total emission rate measured in each survey can be found by comparing the cumulative emission rate of each sample to the model function. Since the model does not extend below the GML FDL due to the onset of sub-unity POD, we read cumulative rates at this threshold (10 kg/h for 150 m sources and 3 kg/h for equipment sources) rather than at the top of the curves. Comparing each sample to the model and its 95% CI, GML is estimated to have measured 91.1% [79.4, 103.4%] of the total emission rate predicted by the model for 150 m sources above 10 kg/h, and CM is estimated to have measured 39.7% [34.6, 45.1%]. For equipment-scale sources, GML measured 103.6% [93.5, 114.2%] of the cumulative emission rate above 3 kg/h. Measured fractions in excess of 100% signify the measured distribution exceeding the joint model distribution over a portion of the model CI, which can be expected from a finite sample size and model fit uncertainty.

In the case of 150 m sources, both CM 2019 and GML appear to have undermeasured the heavy tail compared to the model distribution. This can be seen from the cumulative emission rates falling below the model in Figure 4a. In fact, the CM measured distribution lies outside the estimated sample error, which could be explained by a departure from lognormal behavior above source emission rates of 10^3.4^ kg/h (see Section S6). The lower than expected cumulative emission rate is consistent with a sharp drop in the measured CM 2019 survival function at 10^3.5^ kg/h, as shown in the inset of Figure S8. By comparing the CM measured distribution to the model function in Figure 4a, it can be seen that most of the CM-model error is indeed inherited from emission sources above 10^3.5^ kg/h. This suggests that either the lognormal distribution does not describe the true emission rate distribution above 10^3.5^ kg/h despite working well below it or that the anomaly in CM 2019 data at 10^3.5^ kg/h might be explained by undersampling, systematic error, or failure of the invariance assumptions mentioned in Section S2. In any case, further measurements of the distribution would more clearly resolve this part of the heavy tail and explain the discrepancy.

Without POD compensation, missed emissions below the detection sensitivity of a given campaign raise the apparent threshold responsible for a given share of the total emission rate. For example, according to CM 2019 data alone, 90% of the total emission rate is contributed by facilities with rates above 249 kg/h, whereas in the GML distribution, the 90% facility rate is 16.9 kg/h. In fact, the true 90% threshold will be even lower because emission sources below GML detection sensitivity are underrepresented in the GML dataset.

In addition, spatial aggregation of emission sources shifts (and reshapes) the entire curve to larger emission rates. Comparing the model curves in Figure 4b shows that the 150 m source curve is shifted to the right of the equipment-level source curve by roughly a factor of 3–5 over most of the domain. The 90% threshold for the total detected emission rate shifts from 16.9 to 6.0 kg/h on the GML data traces. The shift toward smaller emission rates can be significant, meaning that measured distributions and sensitivity thresholds should be interpreted at the specified spatial aggregation level and not directly compared at different spatial aggregation levels.

Comparing the CM and GML distributions shows that the total methane emission rate from O&G production infrastructure in the survey region is significantly greater than previously reported, with GML measuring 2.3 times that measured in the CM 2019 campaigns (and also 2.3 times that in the CM 2020–21 campaigns; see Section S9). If observation of emissions is viewed as an ergodic process, then the cumulative emission rate distributions shown in Figures 4 and S11 may be seen as representative of the total average emission rate for production infrastructure in the survey region. In this case, proportions of the total emission rate from the plots can be compared to measurements of regional methane flux. Based on top-down inversion from Tropospheric Monitoring Instrument (TROPOMI) measurements of regional methane flux, Cusworth et al. estimated that sources measured in the CM 2019 survey represent 59% (CM 2020–21: 49%, where fractions for each of the three campaigns are weighted by survey area) of the total methane emission rate in the survey region.^22^ This estimate is somewhat higher than the proportion of cumulative emission rate measured by CM 2019 compared to GML (1/2.3 = 43%), suggesting that the emission rate inferred from TROPOMI in ref (22) underestimates total emissions by 37% (i.e., 59%/43% – 1) if GML and CM campaign data are representative of the same emissions process.

In summary, GML detection data extends the measured emission rate distribution for Permian Basin O&G production infrastructure beyond CM sensitivity limits by roughly 2 orders of magnitude. In joint analysis, intensive sampling of the heavy tail by CM is complemented by GML’s higher detection sensitivity. In the region surveyed, facility-sized emission sources with rates below the CM campaign detection sensitivity (280 kg/h at 50% POD) contribute 67% of the total emission rate from sources with rates above 10 kg/h. The density of these sources and their constituent equipment-size emission sources (at rates above 3 kg/h) was measured without POD degradation by GML. According to the GML sample without POD correction, 90% of the total cumulative emission rate measured originates from equipment-sized sources with rates larger than 6.0 kg/h. This threshold rate would become even smaller if sources below 3 kg/h were measured at their true density rather than at POD < 1. Emissions monitoring campaigns require both high sensitivity and intensive sampling to accurately capture the emissions distribution.

## Supporting Information Available

GML methane emission source detection data at 150 m spatial aggregation (*.csv). GML methane emission source detection data at 2 m spatial aggregation (*.csv). Single-emitter classification of CM 2019 plume images (*.csv). Geospatial polygons bounding GML dataset (*.kml) (ZIP)
Supporting analysis (PDF)
