# Supplementary material for: Extension of Methane Emission Rate Distribution for Permian Basin Oil and Gas Production Infrastructure by Aerial LiDAR
Source: Environ Sci Technol. 2023 Aug 10;57(33):12234–41. doi: 10.1021/acs.est.3c00229 (PMC10448715; doi:10.1021/acs.est.3c00229)
Supplement: Supplementary file 2 — es3c00229_si_002.pdf [file es3c00229_si_002.pdf]

Supporting Information

Extension of Methane Emission Rate Distribution for Permian Basin Oil and Gas Production Infrastructure by Aerial LiDAR

William M. Kunkel,* Asa E. Carre-Burritt, Grant S. Aivazian, Nicholas C. Snow, Jacob T. Harris, Tagert S. Mueller, Peter A. Roos, and Michael J. Thorpe*

Bridger Photonics, Inc., 2310 University Way Bldg 4-4, Bozeman, MT 59715, USA

E-mail: William.Kunkel@bridgerphotonics.com; Mike.Thorpe@bridgerphotonics.com

Summary: 26 pages, 13 figures, 1 table.

Contents

Section S1: Spatial aggregation to 150 m sources **S3**

Section S2: Data preparation and alignment **S4**

S2.1: Sample composition and emitter types included S4

S2.2: Spatial overlap of CM and GML samples S5

S2.3: Temporal overlap of CM and GML samples S6

S2.4: Scan repetitions S7

Section S3: Statistical test on CM and GML distributions **S11**

Section S4: Likelihood function **S13**

Section S5: Akaike information criterion (AIC) analysis **S14**

Section S6: Model fit and scaling **S17**

Section S7: Equipment-scale emission source filtering **S19**

Section S8: Density plots (CM 2019 single emitters, CM 2020-21) **S20**

Section S9: Cumulative emission rate distribution (CM 2020-21) **S21**

Section S10: Monte Carlo estimation of sample error **S23**

Section S11: Exclusion of pipelines from CM dataset **S23**

References **S26**

S1. Spatial aggregation to 150 m sources

Spatial analysis of GML data is performed by first assigning an emission origin point, or "location," to each detected plume. Detections observed at different times are associated with the same location if they are co-located within 2 m. Emission locations were spatially aggregated to 150 m sources by a clustering algorithm that iterates through a list of GML locations to build a temporary table of locations within 150 m to all other locations in the current cluster. After all unclustered locations in the list have been compared to the temporary cluster (sequentially, in fixed arbitrary order), those in the temporary cluster are removed from the waiting list. New clusters are formed in this way until no locations remain in the waiting list.

GML detections can also be aggregated to "facilities" described by polygons enclosing site assets. Facility polygons represent the boundaries around actual groups of surface infrastructure and are usually defined by the facility pad footprint. Polygons can be provided by operators based on site data or generated from aerial photography, in which case the polygon is drawn either manually or by an artificial intelligence model. A mix of AI-generated and manually defined polygons was used in the dataset in this work. A polygon is defined for every facility on a GML flight path regardless of whether an emission is actually detected.

Comparing the number of GML locations in each facility to the number per 150 m diameter source shows a near correspondence between the two aggregation styles (Fig. S1), supporting the use of 150 m aggregation to represent facility-sized sources. A smaller aggregation area (30 meters) displays a steeper roll-off in number of detection locations per source. For GML, the proportion of facilities with at least one detection in the first scan was found to be 38.3% (or 32.9% in the first overflight within the 15-minute scan window described in Sect. S2.4.2), much higher than the reported 1.48% rate[S1] of well sites in the CM 2019 campaign. This difference may be explained by differences in detection sensitivity described in the analysis.

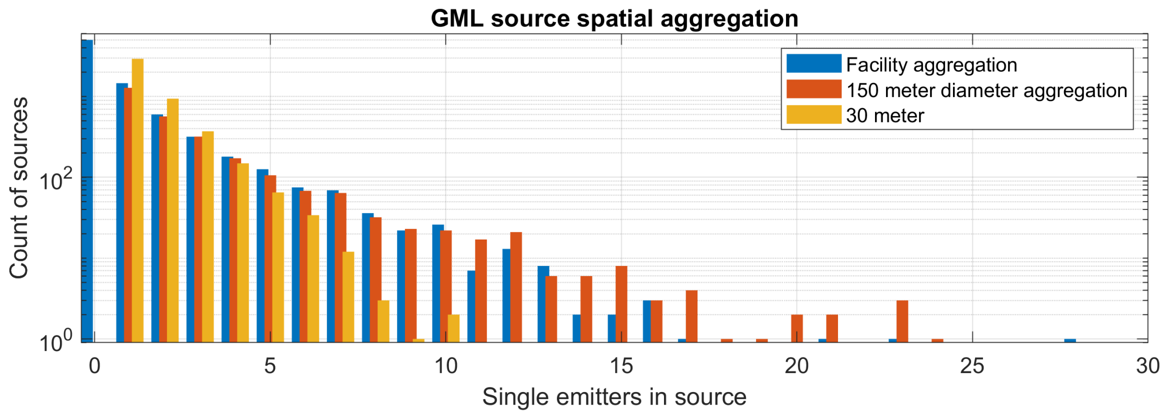

Figure S1: Histogram counting spatially aggregated sources by number of emitters in aggregation area.

S2. Data preparation and alignment

Before jointly analyzing emission detection data from diverse sources, several aspects of data collection and dataset composition must be considered. In this section we aim to address some important aspects of data alignment, specifically (1) compilation of GML survey data and types of emitters included, (2) spatial overlap of CM and GML surveys, (3) temporal overlap of CM and GML surveys, and (4) influence of scan repetitions on reported emission rates. In preparation for this work, efforts were made to directly align the datasets as much as possible. Where datasets do not align, limited assumptions of spatial or temporal invariance are used to set up the analysis. These are pointed out where relevant in the details of the data preparation process below.

S2.1 Sample composition and emitter types included

The GML sample is compiled from sets of anonymized survey data collected under contracts for client O&G operators. Survey sets for compilation were chosen for geographical and temporal overlap with CM data without considering analysis results. Whereas CM campaigns blanketed entire geographic areas, GML surveys were targeted to client facilities. Clients were given advance notice of when scans would occur (typ. accuracy ±2-3 days). The sample is comprised of scans of sites belonging to 28 individual operators.

Sites included in the GML sample were in the O&G production sector and do not include midstream/distribution infrastructure. Types of infrastructure included in the GML sample consist of wells, separators, tanks, compressors, flares, vapor recovery units, generators, and facility piping. Equipment types were not identified at every facility scanned, though this capability is currently under development. In this work, CM data have been filtered to exclude detections from O&G pipelines unless otherwise marked. In the CM 2019 dataset,[S2] measurements with all *source type* tags were included except for "pipeline" and "NA." For CM 2020-21,[S3] the accepted tags were "tank," "well," "compressor," "processing," and "refinery." Exclusion of pipelines seems to have a negligible effect on the shape of the CM distribution, as shown in Sect. S11.

False positive detections can occur in GML detection data, but practically only near the GML detection limit. For emission rates more than a factor of two above the GML detection limit the likelihood of false positives is vanishingly small. GML uses a physics model of the LiDAR measurement noise processes (shot noise, photodetector noise, speckle noise) to estimate the noise on each methane concentration LiDAR measurement based on received light levels. During processing of GML data the signal to noise ratio for each measurement is used in a statistical algorithm to detect regions of elevated methane concentration. The detected regions of elevated concentration are then submitted to emitter analysis, which only assigns an emission if a hot spot in both detection confidence and concentration is detected at the upwind end of the detected plume.

S2.2 Spatial overlap of CM and GML samples

GML and CM 2019 samples were restricted to the GAO coverage polygons in the Delaware and Midland Basins provided in Ref. S1. Geography is shown in Fig. S2. Restriction to the GAO polygons excludes 29 out of 1756 detected facility sources in CM 2019. GML detection locations occupy a subregion of both GAO polygons. We assume that the complementary area in the GAO polygon does not significantly affect the emission rate distribution.

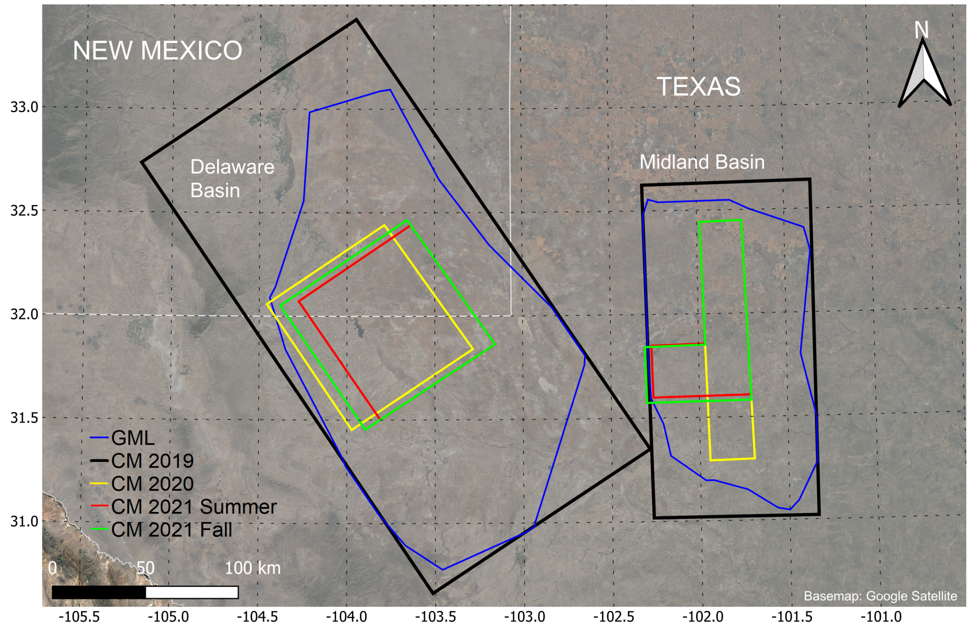

Figure S2: Geospatial domain of GML and CM samples. GML polygons contain all detected emission locations and include a random buffer so vertices do not correspond to detections. CM 2019 polygons reported in Ref. S1 represent areas surveyed at least once by GAO. CM 2020-21 polygons are approximate based on detection coordinates; details reported in Ref. S4.

Though the CM 2020-21 coverage areas[S4] intersect the 2019 GAO polygons, they do not cover the entire area of the 2019 polygons and contain a small amount of additional area outside them. We do not explicitly align GML and CM 2020-21 survey areas in this work, but rather assume that the emission rate distribution is roughly spatially invariant among these areas. We use the same GML dataset for joint analysis with CM 2019 and CM 2020-21. We apply no geographic filters to CM 2020-21 other than to select the campaigns that took place in the Permian Basin (*source ID* markers “F,” “E,” and “J” in the published dataset[S3]).

S2.3 Temporal overlap of CM and GML samples

A timeline of plume detections in the GML and CM measurement campaigns is shown in Fig. S3. GML scans were performed between Jan 2020 and Feb 2022, whereas the CM campaigns took place in Sept-Nov 2019 (CM 2019) and Jul 2020-Nov 2021 (CM 2020-21). Analysis in the Results section assumes stationarity in the shape of the emission rate dis-

tribution with time (i.e. does not change with choice of time origin). However, stationarity of the scale of the distribution is not required. The joint analysis computes separate likelihoods for each dataset and scales the density and cumulative emission rate traces to the total density above the CM full detection limit.

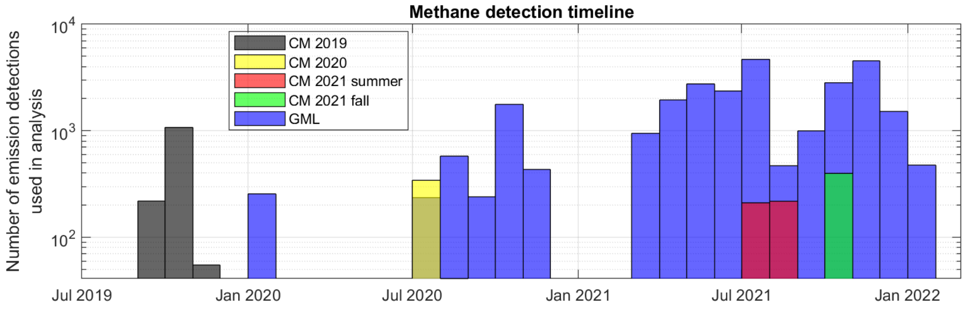

Figure S3: Plume detection counts versus time for CM and GML measurement campaigns.

S2.4 Scan repetitions

CM and GML campaigns were conducted with different approaches to scan repetitions. Number of scans per emission source is shown in Fig. S4. Fewer scans were performed per 150 m source with GML (median: 2 scans) in comparison to CM (2019 median: 6 scans, 2020-21: 4 scans). In CM campaigns, repeated scans over a given source were performed independently of previous results. No minimum number of scans was used to filter the datasets for this work. In GML surveys, repeated scans were performed only on locations where an emission was detected in the first scan. This means that emissions measured by GML were effectively found in just one scan, and repeat measurements were not independent. Most emission sources in the CM campaign had multiple opportunities to be detected, so a greater fraction will have been detected. To address these issues, we describe two solutions below: how to express the distributions in a form that enables direct comparison, and how GML observations are handled considering overflight repetitions and conditionality.

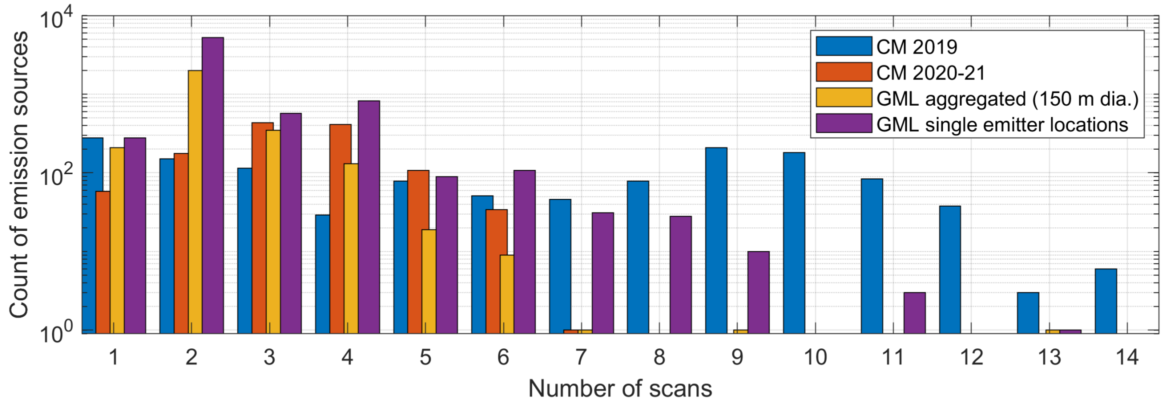

Figure S4: Histogram counting emission sources by number of scan repetitions.

S2.4.1 Transformation to single-scan equivalent

GML and CM distributions are expressed in a "single-scan equivalent" form for alignment. We adopt the notation and terminology of Cusworth et al.[S1] for the persistence-adjusted emission rate $q = f\bar{q}$, where f is the observed persistence $f = M/N$, with M as the number of non-zero unique detections and N as the number of scans, and $\bar{q}$ is the mean of all non-zero measured emission rates

$$\bar{q} = \frac{1}{M}\sum_{i=1}^{M} q_i, \tag{S1}$$

where q_i is a non-zero unique emission rate measurement. For a given source emitting intermittently at a single rate, $\bar{q}$ should be consistent across number of measurement scans, which aids in comparing measurements with different numbers of scans.

To plot emission density and cumulative emission rate on a $\bar{q}$ axis requires further adjustment using the persistence. Consider a point on the detection density function, or rather, a single point in a discrete series representing detection frequency, as shown in Fig. S5a. The persistence adjusted detection frequency (blue), where the emission rate is q, is accessible only from a repetitive sample set and not from a single scan, since the persistence f is needed to obtain q. The detection frequency can be replotted at $\bar{q}$, which effectively removes the persistence from the emission rate. This results in an effectively higher emission rate (green dashed) which overrepresents the density at this emission rate. To obtain a correctly

weighted frequency for summation, or density for integration, the frequency of the source must be reduced by the persistence (red). Where the density or frequency function contains many points, the remapping of q to $\bar{q}$ and the persistence weighting f applies to all points on the curve.

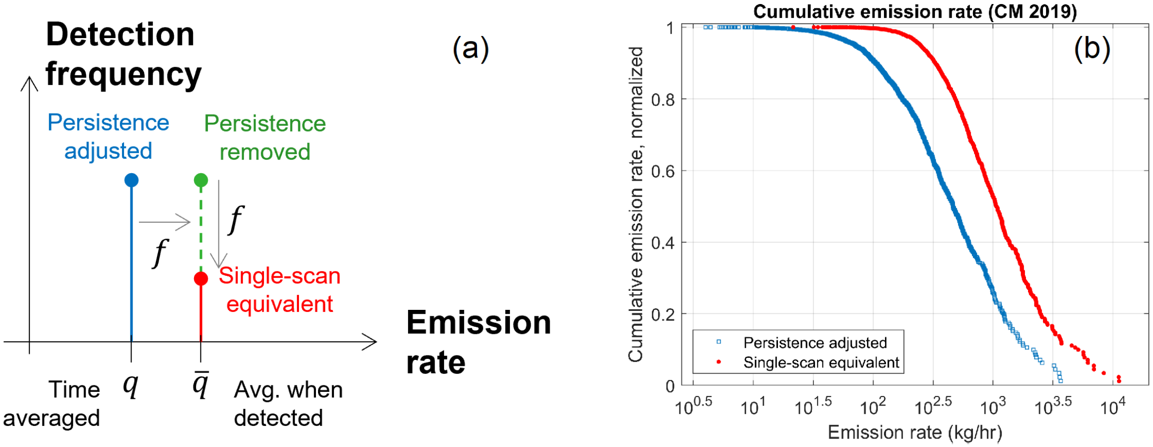

Figure S5: Transformation of emission distribution using the observed persistence f, for (a) a discrete point in a measurement series and (b) the cumulative emission rate distribution (CM 2019, 150 m sources). In both plots, emission rate on the x-axis means either q or $\bar{q}$ as indicated. Distributions shown in (b) follow Eq. S2, reaching the same total emission rate without independent normalization.

Next, consider the implications for the cumulative emission rate distribution. For a finite set of measurements, the cumulative emission rate is computed using the discrete sum

$$c(x) = \frac{1}{\sum q} \cdot \begin{cases} \sum\limits_{q \geq x} q & \text{(persistence adjusted)} \\ \sum\limits_{\bar{q} \geq x} f\bar{q} & \text{(single-scan equivalent)} \end{cases}, \tag{S2}$$

where x is the emission rate. The result of Eq. S2 applied to the CM 2019 sample used for analysis is shown in Fig. S5b. Different forms of the sum yield the same total emission rate since each source contributes the same argument to the sum ($q = f\bar{q}$). Effectively the contributions have been reordered and replotted on the x-axis according to the corresponding value of q or $\bar{q}$. As a result, the single-scan equivalent distribution is a reshaped and horizontally shifted version of the persistence-adjusted distribution.

Although this treatment conveniently transforms distributions for comparison regardless of number of scans, the resulting distributions are not exact. Noting that the observed persistence f is an observation of an event with probability equal to the actual source persistence times the probability of detection (POD), some distortion of the distribution can be expected where sources with POD < 1 from below the FDL are shifted above it. Whereas this affects multi-scan datasets like CM, single-scan datasets (which GML approximates) would not be affected.

S2.4.2 GML detections

GML observations of a given emission source come at three different levels: overflight, location scan, and aggregated source scan. A location scan is comprised of one or more aerial passes ("overflights") of an emission source seen at GML source resolution ($\sim$ 2 m). The first measurement out of all overflights within a 15-minute time window, inclusive of measurements with zero and non-zero emission rates, is selected to represent the emission rate for the scan. Scan measurements are then converted to a persistence-adjusted rate q and associated observed persistence f for the location. These are used to find the "average when detected" rate $\bar{q}$ in the same way as for the CM data, using Eq. S1.

For spatially aggregated sources (150 m), emission rates are found by adding the persistence adjusted emission rates for each location in the source, and dividing by a composite persistence value for the source,

$$f_{\text{agg}} = \frac{\sum_i q_{\text{loc},i} f_{\text{loc},i}}{\sum_i q_{\text{loc},i}}, \tag{S3}$$

where $q_{\text{loc},i}$ is the persistence adjusted emission rate and $f_{\text{loc},i}$ is the observed persistence, where both correspond to the ith location in the source. In other words, the aggregated source persistence is an average of the observed location persistence values, weighted by the persistence-adjusted location emission rates. The average emission rate for the source, when

detected, is then calculated as $\bar{q} = \sum_i q_{\mathrm{loc},i}/f_{\mathrm{agg}}$.

S3. Statistical test on CM and GML distributions

A two-sample Kolmogorov-Smirnov (K-S) test[S5] is used to check for differences between the tails of the GML and CM samples. It serves as a check on the assumption that the CM and GML datasets are well aligned. K-S is a non-parametric test with a standard null hypothesis (no statistically significant difference between the samples). Here the test is performed on the survival function for a single-scan equivalent sample,

$$S(x) = \frac{\sum\limits_{\bar{q} \geq x}^{\infty} f(\bar{q})}{\sum\limits_{\bar{q}=x_L}^{\infty} f(\bar{q})}, \tag{S4}$$

where x is the emission rate and $x \geq x_L$, with x_L as the lower bound of a range of interest, and f is the observed persistence for a given measurement with emission rate $\bar{q}$. The sum is represented as a stepwise function for the K-S test. As mentioned in the Results section, we choose $x_L = 600$ kg/h as the effective full detection limit of CM measurements.

The survival function of the GML and CM 2019 samples are plotted in Fig. S6. The Kolmogorov-Smirnov (K-S) statistic shows the maximum absolute residual between the two sample distributions. The number of measurements in the GML sample is small in this range. In both cases the associated p-values are high and do not indicate rejection of the null hypothesis.

The K-S test is also used to check the measured emission distribution for the CM 2020-21 campaigns against GML. In 2020-21, CM conducted three campaigns around the Midland and Delaware sub-basins (2020 summer, 2021 summer, 2021 fall). Each campaign is smaller than CM 2019 in number of detections (see Fig. S3) and number of overflights. Spatial overlap among these campaigns is partial; overlap with CM 2019 is also partial.[S4] For the analysis in this paper, no controls for spatial overlap were used, under the assumption that

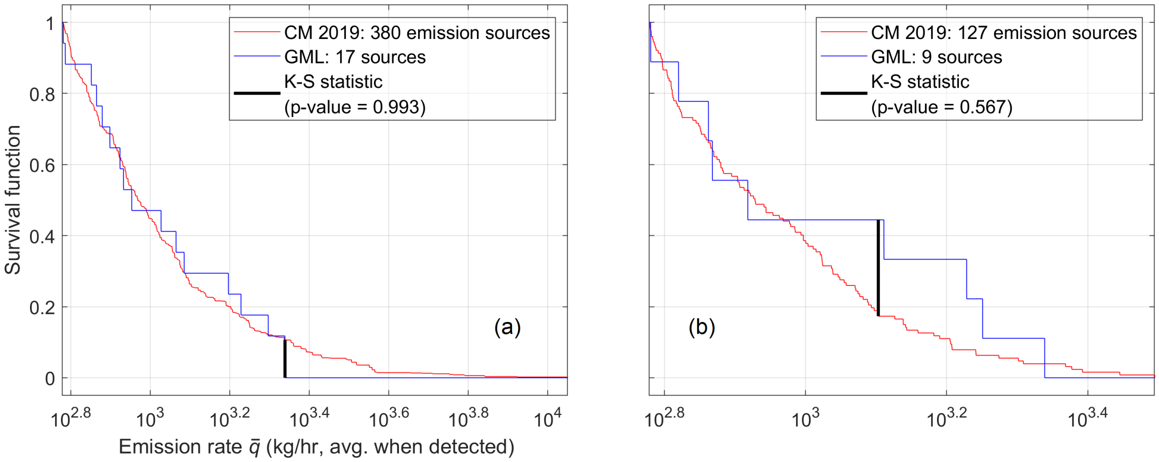

Figure S6: Survival function of single-scan equivalent CM 2019 and GML source detections above 600 kg/h where sources are defined by a (a) 150 m aggregation diameter and (b) single emitter. Kolmogorov-Smirnov (K-S) statistic and associated p-value are shown.

the shape of the emission rate distribution is spatially invariant over the CM 2019 and CM 2020-21 domains. The GML dataset is unchanged whether comparing to CM 2019 or CM 2020-21.

Fig. S7 shows the distributions and K-S test results. For the CM 2020 campaign, a deviation around $10^{2.9}$ kg/h is responsible for a slightly low p-value of 0.166. When grouped with the other campaign data, however, the CM 2020 deviation no longer causes the maximum difference in sample distributions (comparing Fig. S7a and Fig. S7d). For analysis in the rest of this paper, all three CM 2020-21 campaigns were merged into one dataset as shown in Fig. S7d.

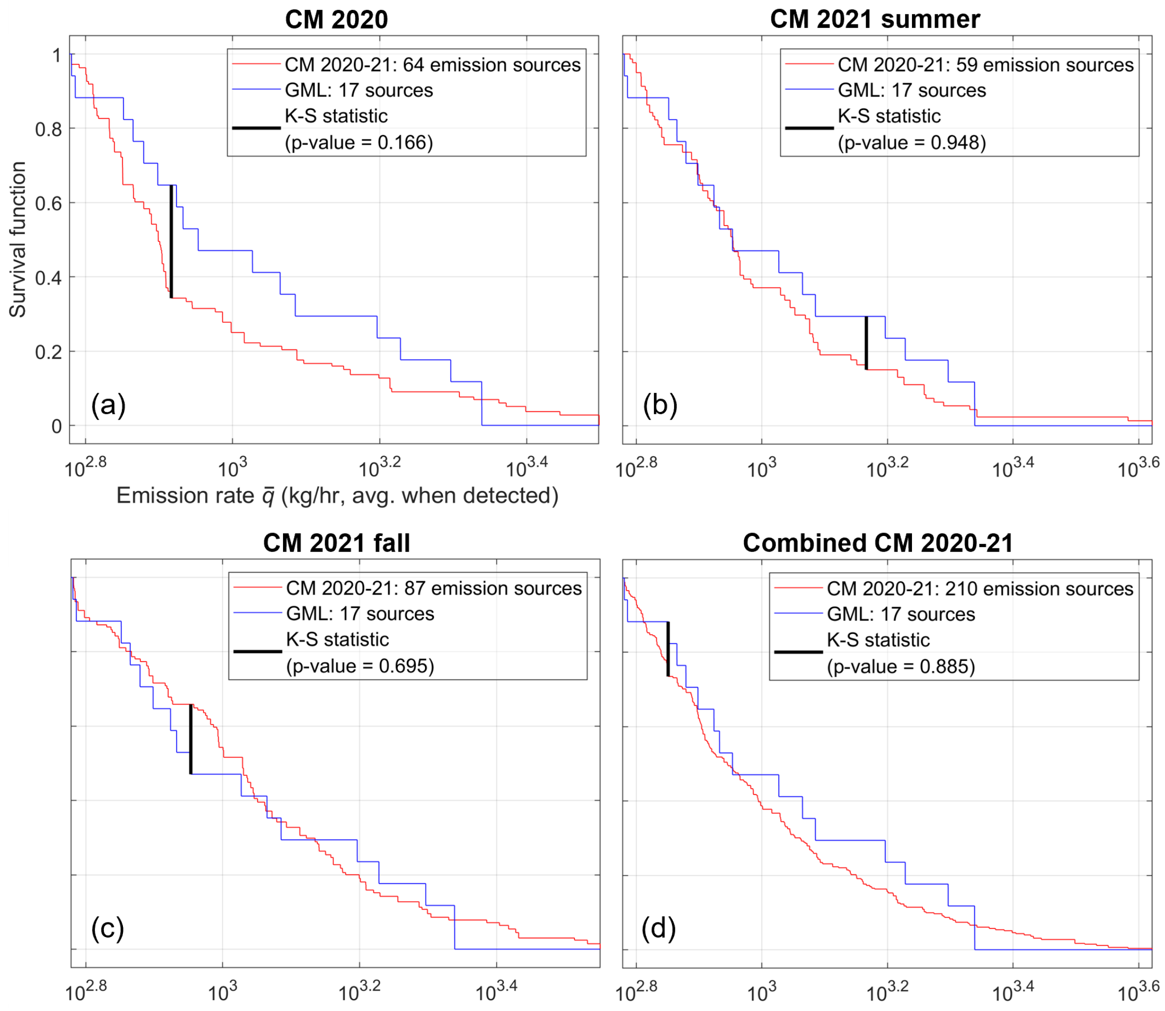

Figure S7: Survival function of CM 2020-21 and GML single-scan equivalent source detections (150 m aggregation diameter) for emission rates above 600 kg/h for campaigns taking place in (a) 2020 summer, (b) 2021 summer, (c) 2021 fall, and (d) all 2020-21 campaigns together. Kolmogorov-Smirnov (K-S) statistic and associated p-value for each case are indicated.

S4. Likelihood function

The likelihood function $L(\theta)$, where θ is the vector of fit parameters, is based on the density function from Eq. 1 normalized to the integration range $x_{L,i} \leq x < \infty$,

$$p_i(x) = \frac{m}{b d_i \Gamma(1/m)} \exp\left(-\left|\frac{(x-x_0)}{b}\right|^m\right), \tag{S5}$$

where $x_{L,i}$ is the FDL, $d_i = 1 - \mathrm{sgn}(x_{L,i} - x_0)\Gamma\left[|(x_{L,i} - x_0)/b|^m, 1/m\right]$, and the subscript i has been added to denote the sample (i.e. GML or CM). Using the standard form for the likelihood function, $L(\theta) = \Pi_{j=1}^{n} p(X_j|\theta)$, where X_j are the observed emission rates in the sample, we obtain the log likelihood function for the ith sample,

$$LL_i(\theta) = \sum_{j=1}^{n} \left[\ln\left(f_{i,j}/\bar{f}_i\right) + \ln\left(\frac{m}{bd_i\Gamma(1/m)}\right) - \left|\frac{X_{i,j} - x_0}{b}\right|^m \right], \tag{S6}$$

where $\bar{f}_i$ is the mean persistence of the sample in the limited domain ($x \geq x_{L,i}$). The term $f_{i,j}/\bar{f}_i$ performs the persistence weighting (vertical part of the density transformation) from persistence-adjusted to single-scan equivalent described in Sect. S2.4.1 while maintaining the property that $\int_{x_L}^{\infty} p(x)dx = 1$.

For joint fits, because the samples are independent, we take the product of likelihoods to obtain the joint log likelihood function

$$LL(\theta) = \sum_{i} LL_i(\theta), \tag{S7}$$

where $i = 1, 2$.

S5. Akaike information criterion (AIC) analysis

AIC analysis was performed on lognormal and generalized lognormal fits to single-sample datasets and joint datasets. Results are shown in Table S1. As seen by the location of AIC minima under single-sample fits where the fit is tested with the same sample in the "1" rows, joint fits do not provide the best representation of each single sample. They instead reduce the joint likelihood of the two independent samples taken together, as seen by the location of joint relative AIC minima under joint fit columns in "2" rows. Models fit to the CM distribution alone tend to have very low values of joint relative likelihood of information loss (see "3" rows), suggesting that models fit to the CM samples alone are not predictive of the

entire distribution through the range over which GML is assessed (≥ 3 kg/h or ≥ 10 kg/h). In addition to the lognormal and generalized lognormal functions shown, log-logistic, Fréchet, Gumbel, and power law model functions were tested but were not optimal in any case.

Table S1: Akaike information criterion (AIC) analysis for different datasets: (a) CM 2019 survey data, 150 m emission sources; (b) CM 2019 survey data, single emitter (or equipment-sized) emission sources; (c) CM 2020-21 survey data, 150 m emission sources. Each table displays (1) AIC values obtained from the likelihood function (parameter values x_0, b, m), (2) relative AIC values $\mathrm{AIC}_{\mathrm{rel},j} = \mathrm{AIC}_{i,j} - \mathrm{AIC}_{\mathrm{min},j}$, where (i, j) signify (sample, fit), and (3) relative joint likelihood of information loss minimization, where the joint likelihood is taken as the product of likelihoods corresponding to each sample, i.e. $\exp[-\sum_j \mathrm{AIC}_{\mathrm{rel},j}/2]$.

(a) CM 2019, 150 m sources

(1)

Fit → ↓ Test	CM Lognorm.	GML Lognorm.	Joint Lognorm.	CM Gen.Logn.	GML Gen.Logn.	Joint Gen.Logn.	Min.
CM	54.0	57.8	55.4	55.9	75.8	57.4	54.0
GML	1873	1080	1081	1112	1081	1083	1080
(Params)	(1.840, 0.880)	(0.880, 1.088)	(0.797, 1.140)	(-4.443, 6.057, 6.224)	(-0.350, 2.167, 3.232)	(0.882, 1.072, 1.929)	

(2)

CM	0	3.8	1.4	1.9	21.9	3.4	
GML	793	0	0.6	32.0	1.4	2.6	
Sum	793	3.8	2.1	33.9	23.3	6.0	2.1

(3)

0	0.41	1	0	0	0.14

(b) CM 2019, single emitter sources

(1)

Fit → ↓ Test	CM Lognorm.	GML Lognorm.	Joint Lognorm.	CM Gen.Logn.	GML Gen.Logn.	Joint Gen.Logn.	Min.
CM	-166	-164	-164	-164	-154	-158	-166
GML	67852	3499	3499	6414	3484	3486	3484
(Params)	(2.346, 0.541)	(0.303, 1.056)	(0.311, 1.052)	(-6.901, 9.355, 16.52)	(0.654, 0.731, 1.531)	(0.629, 0.770, 1.619)	

(2)

CM	0	1.8	1.7	1.4	12.1	7.8	
GML	64336	14.1	14.2	2930	0	1.8	
Sum	64336	15.9	15.9	2931	12.1	9.7	9.7

(3)

0	0.04	0.04	0	0.30	1

(c) CM 2020-21, 150 m sources

(1)

Fit → ↓ Test	CM Lognorm.	GML Lognorm.	Joint Lognorm.	CM Gen.Logn.	GML Gen.Logn.	Joint Gen.Logn.	Min.
CM	-137	-129	-136	-136	-129	-130	-136
GML	14235	1080	1081	1625	1082	1082	1080
(Params)	(2.650, 0.500)	(0.880, 1.088)	(0.928, 1.059)	(-4.713, 7.623, 17.04)	(-0.350, 2.167, 3.232)	(-1.957, 3.599, 4.494)	

(2)

CM	0	7.8	7.1	1.0	7.8	6.9	
GML	13155	0	0.2	544	1.4	1.9	
Sum	13155	7.8	7.4	545	9.2	8.7	7.4

(3)

0	0.81	1	0	0.39	0.50

S6. Model fit and scaling

Results from the fit optimization for CM 2019 (150 m sources) are shown in Fig. S8. Measured data in each survival function are plotted according to Eqn. S4, which scales each sample to $S(x_{L,i}) = 1$ at the respective FDL, $x_{L,i}$, where i denotes the sample. The model function is correspondingly normalized using integrals over the density $p_i(x)$ given by Eqn. S5.

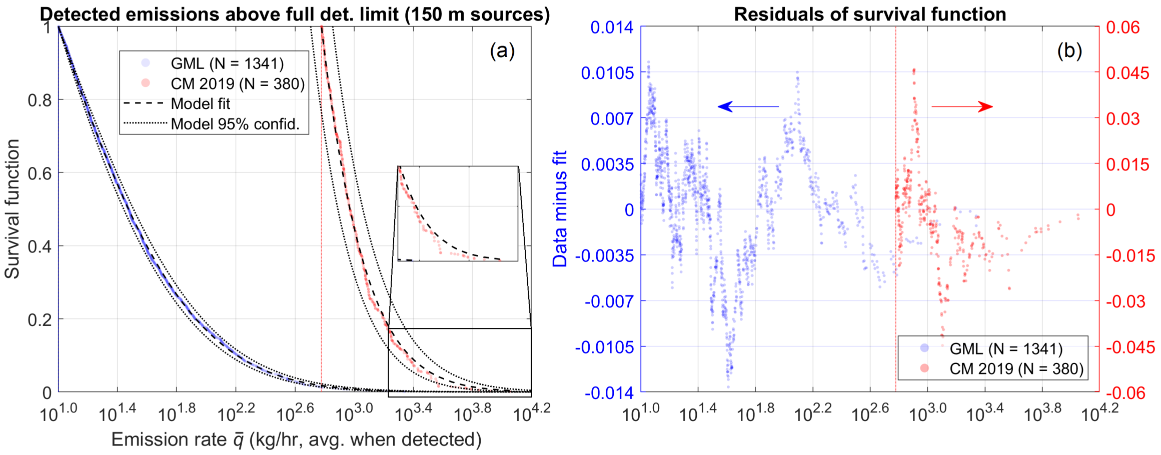

Figure S8: Joint model fitting of 150 m aggregated emission sources showing (a) survival function and (b) fit residuals. Inset: zoomed-in view of largest CM emission rates.

Residuals for both traces show that the survival function crosses the model multiple times without a strong bias toward the positive or negative values. However, CM residuals are negative for emission rates above roughly $10^{3.4}$ kg/h. This does not strongly impact the density function fit, but it does influence sample agreement with the model for cumulative emission rate (i.e. the integral of the density function weighted by emission rate).

When plotted as density functions as in Fig. 2, traces are scaled to a common reference. The factor $1000/n_{\mathrm{CM}}(x > x_{L,\mathrm{CM}})$ is used to scale the CM data to 1000 detections above the CM FDL, where n is the number of detected sources in the specified range. The GML series is scaled by the factor

$$\frac{1000}{n_{\mathrm{GML}}(x > x_{L,\mathrm{GML}})} \frac{1}{\int_{x_{L,\mathrm{CM}}}^{\infty} p_{\mathrm{GML}}(x)}, \tag{S8}$$

where "CM" or "GML" fill in the subscript i in Eqn. S5. The right-hand term of Eqn. S8 rescales the number of detected sources above the GML FDL by the ratio of the survival

functions to each FDL, where both terms in the ratio are evaluated at the CM FDL (that is, recognizing the numerator as $1 = \int_{x_{L,\mathrm{CM}}}^{\infty} p_{\mathrm{CM}}(x)$). These scale factors assume that the size of both samples is sufficiently large above the respective FDL and that sample error in the number of detected sources is negligible. Likewise, the model function is scaled by the factor $1000/\int_{x_{L,\mathrm{CM}}}^{\infty} p_{\mathrm{GML}}(x)$ but with no assumptions about sample size.

Fit results in terms of the survival function are shown for CM 2019 equipment-scale sources and CM 2020-21 150 m sources in Fig. S9. In cases where the CM residuals tend to be negative but GML residuals tend to be positive, the fit is located in between the two samples. To some extent, the model disagrees with CM due to the GML measurements in these cases. Other possible reasons for the fit to be above the CM measurement distribution in the heavy tail include (1) the model functional form or parameter values do not adequately represent the rapid decline in sources in the heavy tail, or (2) the heavy tails measured by CM are reshaped relative to GML by other factors such as quantification bias, such as that reported in Ref. S6. Assuming that the CM and GML measured distributions are in fact aligned, apparent differences may be explained by the heavy tail of the distribution rolling off faster than the model fits above emission rates of roughly $10^{3.4}$ kg/h.

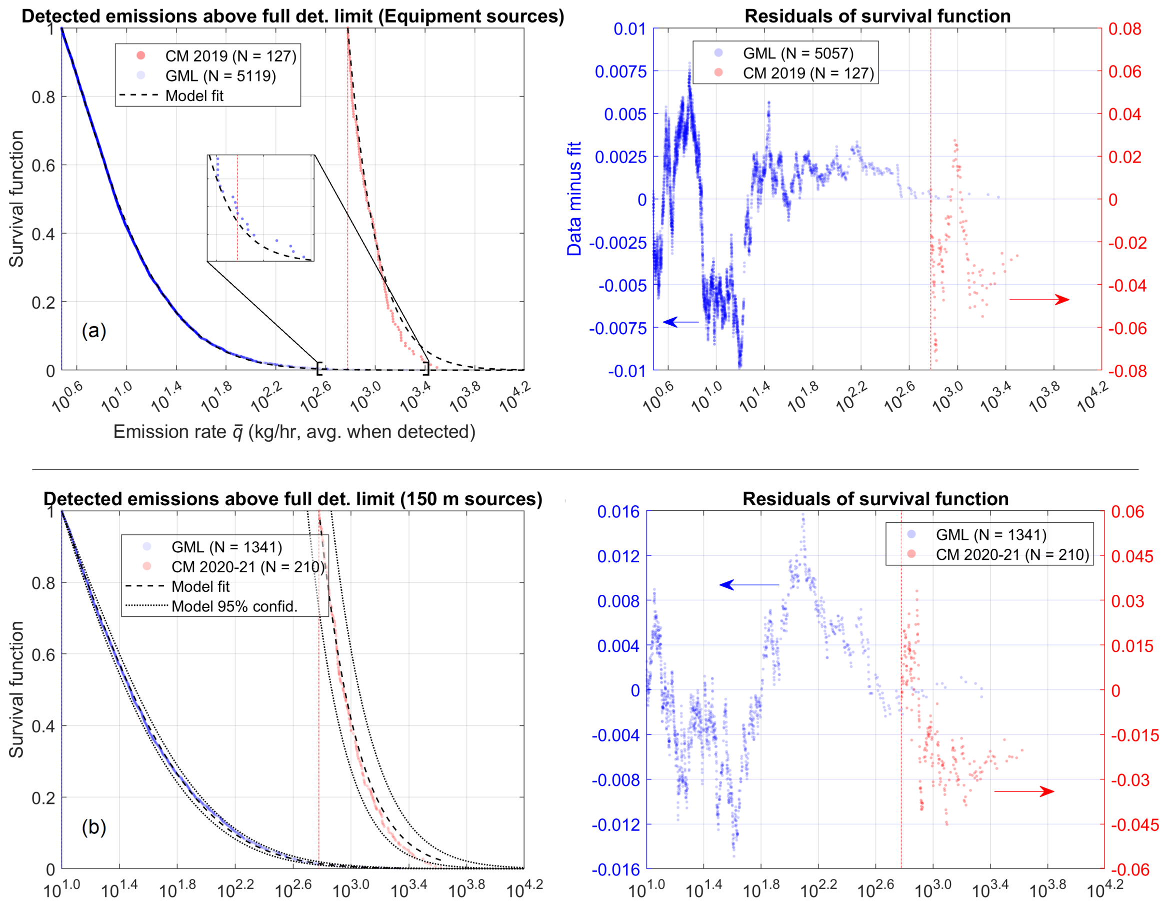

Figure S9: Survival function and residuals showing joint model fitting of GML to (a) CM 2019 equipment-scale sources and (b) CM 2020-21 facility-scale sources (150 m diameter). Inset (a): zoomed-in view of largest GML emission rates.

S7. Equipment-scale emission source filtering

Human analysts classified CM 2019 plume images in a binary fashion as either "single emitter" or "multiple or unclear." Detections classified as "single emitter" were selected for analysis. Scan data were cut to include only the first scan at each source, making the dataset effectively single-scan ($f = 1$). This filter changed the number of CM 2019 sources after other filters (removing pipelines, restricting to GAO survey polygons) from 1348 at 150 m to 645 single emitters. For GML, skipping aggregation increased the number of GML sources from 2727 to 7176, though the number of GML sources above the CM FDL shrank from 17 to 9. Spatial aggregation significantly affects the CM distribution, whereas O&G pipeline sources do not

(Section S11). Fit residuals display similar behavior to those from 150 m sources (Sect. S6).

S8. Density plots (CM 2019 single emitters, CM 2020-21)

Detected emission density from analysis with CM 2019 single emitter and CM 2020-21 samples is shown in Fig. S10. As shown in Fig. S10a, the CM 2019 single emitter sensitivity at 50% POD is seen to be 321 [277, 382] kg/h. This overlaps with the confidence interval of the CM 2019 detection sensitivity at 150 m aggregation. Further details of the single emitter distribution in contrast to the 150 m distribution are described around Fig. 3. For the CM 2020-21 sample, Fig. S10b shows the density with CM 2019 traces reproduced for comparison. The sensitivity at 50% POD is 252 [227, 282] kg/h, which suggests a possible improvement over the CM 2019 campaign sensitivity (possibly from flight altitude; CM 2020-21 campaigns were flown at 4.5 km AGL only). The CM 2020-21 sample is scaled to the CM 2019 sample using the ratio of the GML scale values given in Eqn. S8 from analysis with both CM samples, a value of 0.833. This ensures that the CM traces are scaled to one another such that the GML traces from both analyses coincide exactly. In other words, the CM traces are both scaled to 1000 total CM 2019 detections above the CM FDL using the GML distribution as a common reference.

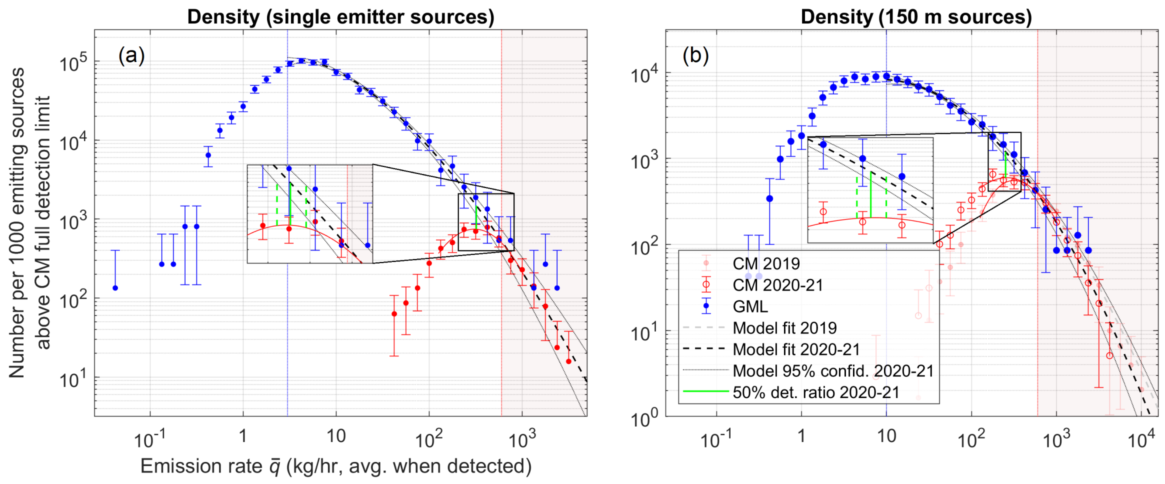

Figure S10: Emission source density from joint analysis with GML for (a) CM 2019 single emitters and (b) CM 2020-21 with 150 m sources. Zoomed in view near the CM sensitivity (insets) shows the 50% detection ratio with respect to model function, along with confidence bounds (dashed green). Model function for CM 2020-21 (150 m) distribution follows Eq. 1 with $m = 2$, $x_0 = 0.928$, $b = 1.059$.

S9. Cumulative emission rate distribution (CM 2020-21)

The CM 2020-21 cumulative emission rate distribution is displayed in Fig. S11. CM 2019 measured data and model function are reproduced in the plot for comparison. Both CM datasets were analyzed jointly with the GML dataset. By comparing the measured distributions at 10 kg/h to the model function and its confidence bounds at this emission rate, we find that CM 2020-21 measured 43.4% [37.8%, 49.2%] of the total cumulative emission rate from 150 m sources above 10 kg/h, whereas GML measured 98.2% [85.5%, 111.3%]. These results are similar to those obtained with CM 2019 data, suggesting consistency between the CM 2019 and 2020-21 campaigns.

Relative scaling of the CM 2020-21 density function to CM 2019 results in the different cumulative emission rate totals shown in the plot. This was performed as described in Sect. S8, where GML density was used as a reference. GML to CM scaling is implied directly from the joint fit without any ad hoc parameters. The ratio of measured totals between the two campaigns (CM 2020-21/CM 2019) is 91% when scaled to one another accounting for

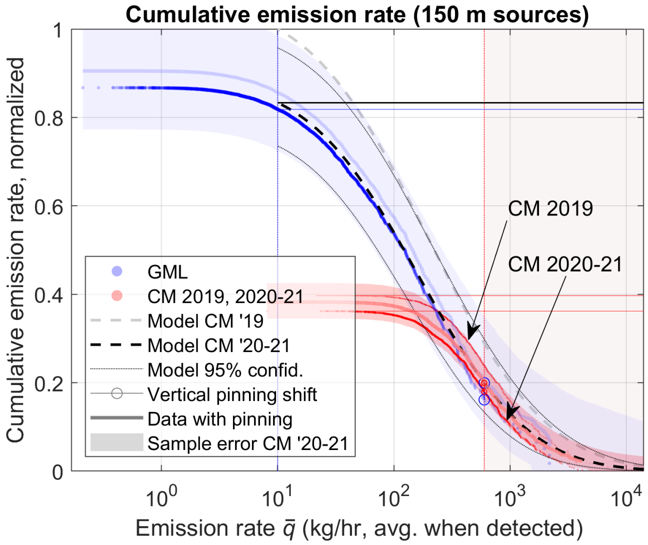

Figure S11: Cumulative emission rate distribution of GML and CM 2020-21 measurements with 150 m diameter aggregated emission sources. Joint GML/CM 2019 model and CM 2019 measured distribution are reproduced for comparison. Distributions from CM 2020-21 joint analysis are scaled to those from the CM 2019 analysis. All traces are normalized to equivalent campaign scale (spatial area, number of overflights). Vertically shifted copies of measured data pinned to the value of the model distribution at the CM FDL guide the eye to suggest the shape of the measured distribution supposing sample error above the CM FDL were suppressed.

sample size and number of overflights, using GML as a reference.

S10. Monte Carlo estimation of sample error

A Monte Carlo algorithm is used to obtain percentile ranges on the cumulative emission rate as a function of source emission rate. New samples are synthesized from the joint best-fit density function at emission rates above the respective FDL. Size of synthesized samples matches the number of detected sources above the FDL in the measured samples. Since the density function expresses the single-scan equivalent, the number of overflights is one for each synthesized detection. For each of n_{MC} =10,000 Monte Carlo trials, a vector of $n(x < x_{L,i})$ random numbers uniformly distributed on the interval $(0, 1)$ is generated. The random numbers are input as arguments to the inverse of the survival function on the domain above the FDL to generate source emission rates. Cumulative emission rate versus source emission rate is calculated from each Monte Carlo trial. Percentiles are found from the set of synthesized Monte Carlo trials on a grid of source emission rates.

Simulated sample error supports the emission rate domain down to each respective FDL. Sample error below the FDL is represented by assuming the same cumulative emission rate increase as the measured sample, with no additional error contributed by samples below the FDL.

S11. Exclusion of pipelines from CM dataset

Exclusion of O&G pipeline sources in the CM 2019 sample produces negligible change in the survival function. By comparison, the effect of filtering the data to single emitter sources changes the distribution significantly. Fig. S12 shows the survival function for "with pipeline" and "without pipeline" filters for 150 m and single emitter aggregation styles. Moderate p-values, and hence no statistically significant difference, are seen between the distributions including or excluding pipelines within each aggregation style. However, the p-value for

a comparison across aggregation styles is outside the 95% confidence interval ($p < 0.05$), indicating that those distributions differ significantly.

Despite the lack of significant change in the shape of the CM 2019 distribution with the inclusion or exclusion of pipelines, small differences around the detection roll-off (300 kg/h) lead to slightly different estimates of the detection sensitivity. Density functions for both aggregation styles with and without pipelines are shown in Fig. S13. Comparing the model bounds at 95% confidence to respective cubic polynomial roll-off fits yields detection sensitivity intervals of 233-279 (with pipelines) and 256-309 kg/h (no pipelines) for 150 m sources, and 258-356 and 277-382 kg/h, respectively, for single emitter sources. Since these intervals overlap significantly, the detection sensitivity roll-off can be considered as weakly dependent on both types of data filter.

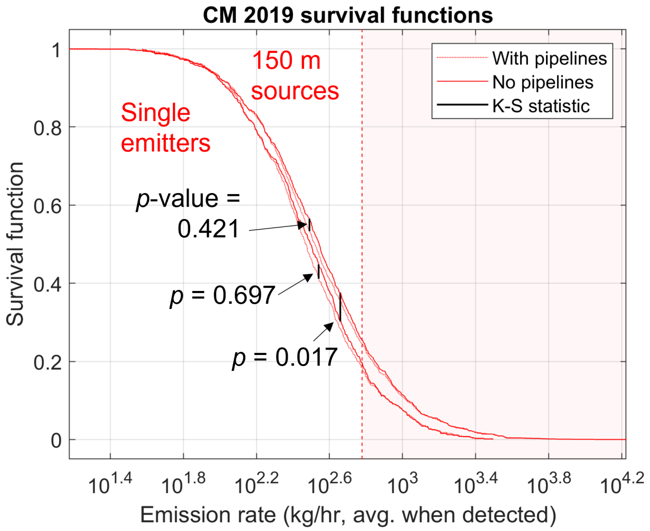

Figure S12: Comparison of CM 2019 survival functions over the range of emission rates in the sample. Data are filtered to either include or exclude O&G pipeline emission sources at both 150 m aggregation and single emitter sources. p-values are indicated for "with pipeline" and "without pipeline" comparisons within each source type and a "no pipeline" comparison across the two source types.

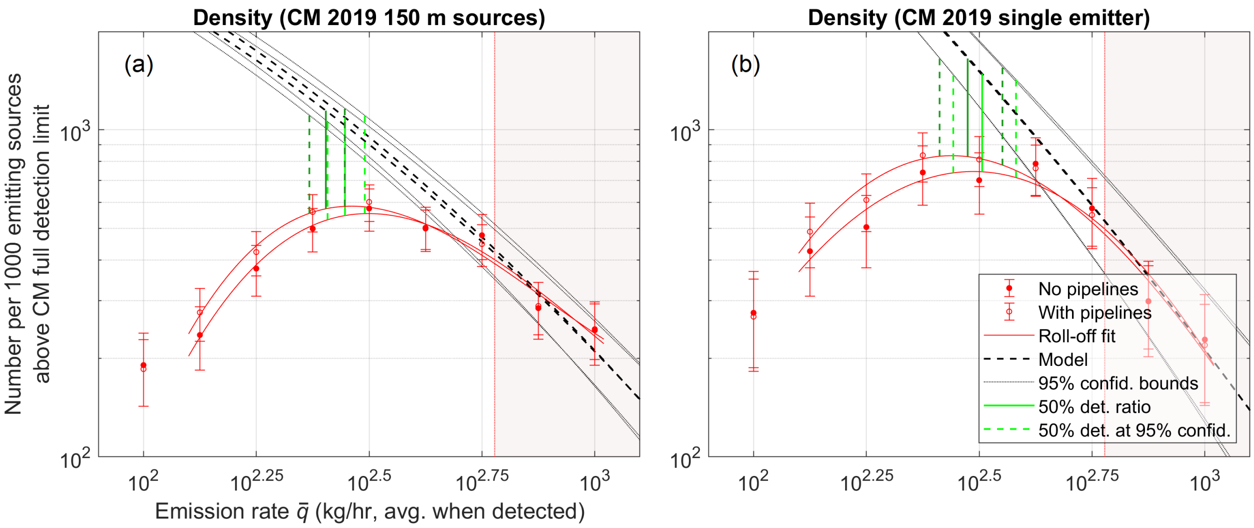

Figure S13: CM 2019 detected emission density plots showing influence of O&G pipeline sources on emission distribution around detection roll-off for 150 m aggregated sources (a) and single emitter sources (b). Model functions are reproduced from joint analysis with GML.

References

[S1] Cusworth, D. H.; Duren, R. M.; Thorpe, A. K.; Olson-Duvall, W.; Heckler, J.; Chapman, J. W.; Eastwood, M. L.; Helmlinger, M. C.; Green, R. O.; Asner, G. P.; Dennison, P. E.; Miller, C. E. Intermittency of Large Methane Emitters in the Permian Basin. *Environmental Science and Technology Letters* **2021**, *8*, 567–573.

[S2] Cusworth, D. Methane plumes for NASA/JPL/UArizona/ASU Sep-Nov 2019 Permian campaign. 2021; [Data set]. Zenodo. https://doi.org/10.5281/zenodo.5610307 (retrieved 27-Apr-2022).

[S3] Cusworth, D. Methane plumes from airborne surveys. 2021; [Data set]. Zenodo. https://zenodo.org/record/7072824#.ZELKvM7MKUm (retrieved 23-Apr-2023).

[S4] Cusworth, D. H.; Thorpe, A. K.; Ayasse, A. K.; Stepp, D.; Heckler, J.; Asner, G. P.; Miller, C. E.; Yadav, V.; Chapman, J. W.; Eastwood, M. L.; Green, R. O.; Hmiel, B.; Lyon, D. R.; Duren, R. M. Strong methane point sources contribute a disproportionate

fraction of total emissions across multiple basins in the United States. *Proceedings of the National Academy of Sciences* **2022**, *119*, e2202338119.

[S5] Bartoszyński, R.; Niewiadomska-Bugaj, M. *Probability and Statistical Inference*; Wiley, 1996; Chapter 16.

[S6] Rutherford, J. S.; Sherwin, E. D.; Chen, Y.; Aminfard, S.; Brandt, A. R. Evaluating methane emission quantification performance and uncertainty of aerial technologies via high-volume single-blind controlled releases. Preprint, https://doi.org/10.31223/X5KQ0X.
